# Insights from *Bacteroides* Species in Children with Type 1 Diabetes

**DOI:** 10.3390/microorganisms9071436

**Published:** 2021-07-02

**Authors:** José Matos, Isabel Matos, Manuela Calha, Pedro Santos, Isabel Duarte, Yameric Cardoso, Maria Leonor Faleiro

**Affiliations:** 1 Faculdade de Ciências e Tecnologia, C8, Campus de Gambelas, Universidade do Algarve, 8005-139 Faro, Portugal; jose.j.matos@gmail.com (J.M.); isabel.csc.matos@gmail.com (I.M.); pedroeenterprise@gmail.com (P.S.); yamericsilva@gmail.com (Y.C.); 2 Algarve Biomedical Center, Research Institute, 8005-139 Faro, Portugal; 3 Unidade de Diabetologia, Centro Hospitalar Universitário do Algarve, 8000-386 Faro, Portugal; mmcalha@gmail.com; 4 CINTESIS—Center for Health Technology and Services Research, Universidade do Algarve, 8005-139 Faro, Portugal; giduarte@ualg.pt; 5 Champalimaud Research Program, Champalimaud Centre for the Unknown, 1400-038 Lisbon, Portugal

**Keywords:** type 1 diabetes, *Phocaeicola dorei*, *Parabacteroides distasonis*, *Bacteroides uniformis*, invasion, phage, molecular mimicry

## Abstract

In our previous study the enrichment of the intestinal proteome of type 1 diabetes (T1D) children with *Bacteroides* proteins was observed, which led us to our current study that aimed to isolate and characterize *Bacteroides* species from fecal samples of T1D and control children. Repetitive sequence-based PCR (rep-PCR) was used for typing the isolated *Bacteroides* species. The antibiotic susceptibility and mucinolytic activity of the isolates was determined. The quantification of specific bacterial groups in the fecal samples was determined by qPCR. The ability to adhere and invade the human colonic cell line HT29-MTX-E12 of strains of *P. dorei*, *B. uniformis* and *P. distasonis* was determined and their whole genome sequencing was performed. The results showed similar numbers of *Bacteroides* species in T1D and control samples, but unique *Bacteroides* species and a higher recovery of *P. distasonis* from T1D samples was observed. Rep-PCR grouped the different *Bacteroides* species, but no discrimination by origin was achieved. T1D children showed a significant increase in *Proteobacteria* and a depletion in *Lactobacillus* sp. All tested *P. dorei*, *B. uniformis* and *P. distasonis* were able to adhere to HT29-MTX-E12 cells but significant differences (*p* < 0.05) in the ability to invade was observed. The highest ability to invade was exhibited by *P. distasonis* PtF D14MH1 and *P. dorei* PtFD16P1, while *B. uniformis* strains were unable to invade. The damage to tight junctions was also observed. The presence of *Lactobacillus* sp. inhibited the invasion ability of *P. distasonis* PtF D14MH1 but not *P. dorei* PtFD16P1. Sequences of agonist peptides of the human natural preproinsulin and the insulin B chain insB:9-23 peptide mimics were identified. The results reported in our study stresses the continued efforts required to clarify the link between T1D and gut microbiota.

## 1. Introduction

Type 1 diabetes (T1D) is characterized by a gradual pancreatic β-cell destruction culminating with insulin deficiency causing a hyperglycemic state, which in the medium- to long-term will cause vascular damage (neuropathy), affecting vision (retinopathy) and kidney function (nephropathy). The burden of genetic heritage in T1D is well recognized. However, the increase in incidence cannot be explained only by a genetic boost. The probability that environmental factors contribute to trigger the disease is currently gaining broader acceptance [1,2]. Among the environmental factors that may be implicated in this triggering event are the exposure of genetic susceptible individuals at early age to gluten containing food, viral upper respiratory tract infections, and exposure to deficient innate immune activator [3,4,5,6].

The disturbance of the gut microbiome in children at risk of developing T1D has been extensively investigated worldwide [7,8,9,10,11,12,13,14,15,16]. In contrast the microbiome of children with established diabetes is still poorly studied [7,14,17,18,19,20]. In the study of Alkanani et al. [7] the gut microbiome of new-onset patients and seropositive subjects from USA with two to four autoantibodies showed a higher (*p* = 0.01) median percentage abundance of *Bacteroides* (35.62%) (21 subjects in the group of new-onset patients, and 16 in the seropositive group) in comparison with individuals with just one autoantibody (6.37), (12 subjects in the new-onset patients group, and five in the seropositive group). Additionally, De Goffau et al. [21] reported higher abundance of the genus *Bacteroides* in children with β-cell autoimmunity who participated in the Finnish Dietary Intervention trial for Prevention of Type 1 Diabetes (FINDIA), and the Trial to Reduce IDMM in the Genetically at Risk (TRIGR) studies performed in Finland. The study conducted by Murri et al. [17] showed that Spanish children with established T1D carried more *Bacteroidetes* in comparison with healthy children. The higher abundance of sequences belonging to *Bacteroides* in the gut of Spanish children with T1D was also reported in the study of Leiva-Gea et al. [20], where they observed an enrichment of 72.21% in T1D cases in contrast to healthy controls that reached 58.45%.

Geographical factors may considerably influence the gut microbiome [12]. For example, in Finland, in the city of Turku, it was reported that *Bacteroides dorei* (currently classified as *Phocaeicola dorei* [22]), dominates the gut of children at risk of developing T1D [8], whereas in the cities of Tampere and Oulu the increase of *B. dorei* was not observed [23]. Instead, these authors observed a dominant OTU, grouping *B. dorei* and *B. vulgatus*, that were inversely associated with following islet autoimmunity. Furthermore, in their study another *Bacteroides* sp., *B. caccae* was inversely associated with islet autoimmunity [23]. The control children in the Cinek et al. study [23] were enriched with an OTU assigned to *Bifidobacterium bifidum*. Another example of geographical differences regarding the composition of T1D children microbiome is the study conducted in the Northern area of China where children newly diagnosed with T1D (<6 months) showed an intestinal microbiota enriched with the genus *Blautia* that was also correlated with the levels of HbA1c, T1D autoantibodies, and titers of tyrosine phosphatase autoantibodies (IA-2) [19]. Furthermore, in the study of Kostic et al. [13] it was reported that the abundance of the genus *Blautia* was correlated with serum glucose levels in children with T1D.

Portuguese children with established T1D showed a metaproteome enriched with proteins originated from *Eubacterium rectale* (currently *Agathobacter rectalis*), *Faecalibacterium prausnitzii*, *B. dorei* and *B. uniformis*, in contrast with the control metaproteome that was enriched in proteins from *Bifidobacterium adolescentis*, *B. longum* subsp. *infantis*, *Ruminococcus*, *Collinsella aerofaciens*, *Coprococcus comes*, and *Clostridium* sp. [18]. However, the qPCR evaluation of *Bacteroides* sp. and *Bifidobacterium* sp. were similar between case and control children, suggesting that the intestinal environment was not affecting the bacterial population, instead modulating its proteome [18]. Altogether, it is important to stress that the gut microbiota in children, either at risk of T1D or with established T1D, shows a dysbiosis, and the genus *Bacteroides* may have some role in the development of the disease.

This study aimed to characterize *Bacteroides* species from fecal samples of T1D children and control children from Portugal (Algarve region), and analyze their characteristics regarding antibiotic susceptibility, mucinolytic activity, its potential to disturb intestinal epithelial cells, and also analyze their genomic characteristics that may be linked with the development of T1D.

## 2. Materials and Methods

### 2.1. Fecal Samples

Seventeen fecal samples from T1D children, aged 9.53 ± 1.88 were obtained (eight female and seven male). The control group included 15 healthy T1D siblings and children with no T1D history in the immediate family, aged 8.20 ± 3.00. All children followed a regular diet (>50% of meat, eggs, vegetables, fruit and milk of regular origin). For the majority of children, sports were practiced twice a week or three times a week, only four T1D children reported not to practice any sports. T1D children were medicated exclusively with insulin. The haemoglobin level (HbA1c) in diabetic children was 8.76 ± 1.50. Samples from each child were collected, immediately transported to the laboratory in cold storage conditions, and directly surveyed. Antibiotic treatment, diagnosis of infectious diseases, and hospitalization up to 3 months before the start of the study were exclusion criteria.

The current study was approved by the Ethics Committee of the Centro Hospitalar Universitario do Algarve, and informed consent was obtained from the parents of the children enrolled in the study.

### 2.2. Bacteria and Culture Conditions

To isolate *Bacteroides* species from fecal samples, the culture medium bacteroides vulgatus selective agar (BVSA) was used [24]. About 0.2–0.3 g of stool specimen was collected to a sterile 2 mL eppendorf, and the feces were resuspended in 1 mL of Phosphate Buffered Saline (PBS). After homogenization, each sample was centrifuged at low speed, 700× *g* for 5 min. Next, 500 μL of the supernatant was collected to a new eppendorf and serial dilutions were prepared. A volume of 100 μL of the dilutions 10^−3^ and 10^−4^ were inoculated in BVSA, and the incubation was achieved using an anaerobic jar with an anaerobic sachet (AnaeroGen, Oxoid, Basingstock, UK) at 37 °C for 3–4 days. Afterwards, characteristic colonies (black, shiny colonies with a dark halo zone) were counted, and representative colonies of each morphological group were transferred to brain–heart infusion (VWR) supplemented with hemin (0.1%, *v*/*v*) and l-cysteine (0.1%, *w*/*v*, Sigma-Aldrich, St. Louis, MO, USA) (BHI + H) to obtain pure cultures. The incubation was conducted at 37 °C for 48 h. Pure cultures were maintained in BHI supplemented with 25% (*v*/*v*) glycerol at −80 °C.

### 2.3. DNA Extraction

The extraction of DNA from stool samples was performed using the kit QIAmp DNA Stool Mini Kit (QIAGEN, Hilden, Germany) according to the manufacturer’s instructions. The stool quantity used for DNA extraction was 220 mg. The extracted DNA was eluted in 200 µL of AE buffer and maintained at −20 °C until use.

The DNA from bacterial cultures was extracted using the Wizard Genomic DNA Purification Kit (PROMEGA) or the GES method [25].

### 2.4. Identification of Bacteroides Species by 16S rRNA Gene Sequencing

Molecular identification of *Bacteroides* species was performed by sequencing the *16S rRNA* gene using the primers pair 27F and 1492R [26]. The *Bacteroides* isolates were cultured in BHI + H for 48 h at 37 °C under anaerobic conditions. Genomic DNA was extracted as described above. The polymerase chain reactions (PCR) amplifications were carried out with the DFS-Taq DNA Polymerase (Bioron GmbH, Römerberg, Germany). Purified PCR products were sequenced with the 1492R primer [27] at the Molecular Biology laboratory of the Centro de Ciências do Mar (CCMar) using standard Sanger sequencing procedures.

The *16S rRNA* gene sequences were analyzed, and those ≥1200 bp were selected for further analysis. The editing of the sequences was conducted using Bioedit (version 7.2.5). The identification of the closest matches to sequence queries was performed using the Blast algorithm of the National Center for Biotechnology Information (NCBI) [27]. The Ribosomal Database Project [28] was also used for the determination of the isolates at the species/strain level.

### 2.5. Characterization of Bacteroides Species by Repetitive Sequence-Based PCR

The *Bacteroides* isolates were analyzed by rep-PCR with *BOX A1R* primer and *ERIC1R* and *ERIC2* primers [29,30]. The PCR reactions were performed with Platinum Taq polymerase (Invitrogen, Eugene, OR, USA).

The PCR amplicons (25 μL) were separated by gel electrophoresis on 1.5% Agarose (Lonza, Risingevej, Denmark) gel in TAE 1× buffer at 100 V during 6 h. The 100 and 500 bp markers (Invitrogen) were used. Gels were stained with ethidium bromide for 30 min and visualized under UV light using the DC 290 Kodac camera.

Analysis of the gel banding patterns was performed using GelCompare II (Biomérieux, Lisbon, Portugal) and dendograms were generated using Pearson correlation similarity coefficient with optimization of 1%, and the cluster analysis was performed using the unweighted pair group method using arithmetic averages (UPGMA) clustering method.

### 2.6. qPCR

The quantification of specific bacterial groups (*Firmicutes*, *Bacteroidetes*, *Bacteroides*, *P. dorei*, *Bifidobacterium* sp., *Clostridium* cluster IV, *Enterococcus* sp., *Lactobacillus* sp. *Proteobacteria* and *Prevotella* sp.) was determined by quantitative real-time PCR (qPCR). The specific primers used are indicated in Table S1. Reactions were performed using SsoFastTM kit Eva Green Supermix (BioRad, Hercules, CA, USA), 0.5 μM of forward and reverse primer and 100 ng of DNA on a CFX96 qPCR thermal cycler (Bio-Rad Laboratories, Hercules, CA, USA). All PCR reactions were carried out in duplicate, and the PCR conditions are shown in Table S2. Reactions containing all the components except template DNA were included as negative controls. The denaturation step occurred at 95 °C, for 10 min. The number of cycles was identical for all target groups (45 cycles). The melting curve was performed by increasing the temperature 0.5 °C each 2 s from 72 °C to 95 °C.

Standard curves were generated using plasmid DNA (pCR ™ 2.1-TOPO) with inserted *16SrRNA* gene of each bacterium. The cloning process was achieved using competent *Escherichia coli* MACH1 (Invitrogen) following the instructions of the vector manufacturer (Invitrogen). Four replicates of the plasmid DNA dilutions in order to obtain the number of copies that ranged from 10^10^–10 were prepared. Data are expressed as Log_10_ DNA copies/g feces.

### 2.7. Antibiotic Susceptibility

The determination of antibiotic susceptibility was achieved as previously described [31,32]. Briefly, *Bacteroides* strains were grown on BHI + H and incubated in an Anaerobic jar (AnaeroGen, Oxoid) at 37 °C for 24 h. Bacterial suspensions were prepared in 0.9% saline to a density of McFarland 1. The disc diffusion assays were performed using Brucella blood agar plates supplemented with hemin (0.1%) and vitamin K1 (1%). The antibiotic discs (Oxoid) were amoxycillin–clavulanate (20/10 μg) (AMC), clindamycin (10 μg) (DA), imipinem (10 μg) (IMP), moxifloxacin (5 μg) (MXF), piperacillin-tazobactam (30/6 μg) (TZP) and metronidazole (5 μg) (MTZ). The plates were incubated in anaerobic conditions at 37 °C for 24 h. Six discs (three per plate) were used. The susceptibility zone diameter provisional breakpoints previously suggested [31,32] were followed: amoxycillin–clavulanate (≥15 mm), clindamycin (≥25 mm), imipenem (≥29 mm), moxifloxacin (≥19 mm), piperacillin-tazobactam (≥25 mm) and metronidazole (≥24 mm). Next, the results for Imipinem and Metronidazole were confirmed by Epsilon test according to manufacturer’s procedure (Biomérieux, Portugal). The breakpoints were established according to EUCAST [33].

### 2.8. Mucinolytic Activity

The ability of several strains of *P. dorei* isolated from T1D and control children to use mucin as sole carbon source was evaluated by growing the bacterial strains in a defined medium [24] supplemented with 1% porcine gastric mucin (PGM) (Sigma, Madrid, Spain) previously dialyzed with SnakeSkin^®^ Dialysis Tubing membrane (cut off: 10 kDa) at 4 °C. As control the bacterial growth was performed in the mentioned defined medium supplemented with glucose as sole carbon source (0.5%, *w*/*v*) and in BHI + H medium. The growth was followed by changes in optical densities (OD_600 nm_) in microplates. For this 180 μL of medium were distributed in each well, and each well was inoculated with 20 μL of the bacterial culture previously grown in the tested medium for 24–48 h in anaerobic conditions. Wells with non-inoculated medium were used as blanks. To assure anaerobic conditions, 30 μL of sterile paraffin oil were added into each well, and the incubation was performed in an anaerobe jar (BioMérieux) with an anaerobic sachet (Oxoid) at 37 ° C for 48 h. The optical readings were performed on a microplate reader (Tecan Infinite M200). Four biological replicates were used. The reference strain of *P. dorei* DSM 17855 and the strain of *P. vulgatus* PtF D2P1 were included.

The mucinolytic activity was evaluated as described by Yesilkaya et al. [34]. The bacterial strains were grown in Defined medium with 1% (*w*/*v*) mucin and 1% agar (DMa). As control, non-inoculated DMa plates were used. The inoculated culture plates were incubated in anaerobic jars during 48 h at 37 °C. Next, the culture plates were stained with 0.1% (*w*/*v*) black amid in acetic acid 3.5 M for 30 min and were decolorized with acetic acid 1.2 M. *Escherichia coli* D3 (isolated from a T1D child) and *E. coli* C1 (isolated from a healthy child) were included as controls. Mucinolytic activity zones were observed around the bacterial growth.

### 2.9. Whole Genome Sequencing

Genomic DNA of strains *P. dorei* PtFD1P5, PtFD8M1, PtFD16P1, PtFD16M14, PtFSb6, PtFSb8, PtFC1P2, *B. uniformis* PtFD3Pch2, PtFSb3P5 and *P. distasonis* PtFD14MH1 for whole genome sequencing was isolated from 2 mL of the bacterial culture prepared as described above. The DNA was extracted using Wizard Genomic DNA Purification Kit (Promega, Madison, WI, USA), according to the manufacturer’s protocol with a modification that included an additional step of lysis with sterile acid-washed glass beads (425–600 µm) (Sigma Life Science, Saint Louis, MO, USA) comprising 1/3 of the volume of the bacterial pellet. The mixture was sonicated for 1 min using the bead beater (Next Advance, Troy, NY, USA).

DNA integrity was analyzed by electrophoresis using a 0.75% (*w*/*v*) agarose gel (Lonza, Copenhagen, Denmark). DNA quantification was performed by using the fluorescence-based Qubit dsDNA BR Assay Kit (Invitrogen, Eugene, OR, USA). Each bacterial genomic DNA was dissolved in 10 mM Tris-HCl buffer at concentrations ranging from 2.7 to 5.0 μg/μL for Standard Whole Genome Service by Illumina next-generation sequencing provided by MicrobesNG (http://www.microbesng.uk, accessed on 2 October 2018) which is supported by the BBSRC (grant number BB/L024209/1). At Microbes NG Genomic DNA libraries were prepared using the Nextera XT Library Prep Kit (Illumina, San Diego, CA, USA) following the manufacturer’s protocol with minor modifications, namely input DNA was increased 2-fold, and PCR elongation time increased to 45 s. DNA quantification and library preparation were performed on a Hamilton Microlab STAR automated liquid handling system (Hamilton Bonaduz AG, Bonaduz, Switzerland). Pooled libraries were quantified using the Kapa Biosystems Library Quantification Kit for Illumina. Libraries were sequenced using Illumina sequencers (HiSeq) using a 250 bp paired end protocol.

Reads were adapter trimmed using Trimmomatic 0.30 with a sliding window quality cutoff of Q15 [35]. De novo assembly was performed using SPAdes version 3.7 [36], and contigs were annotated using Prokka 1.11 [37]. The scaffolding of the contigs was achieved using scaffold builder [38].

The circular genome maps were generated using CGView server Beta (version 1.7) [39]. The tracks GC Skrew, GC Content and Prokka Annotation were added [37]. In Prokka Annotation the default parameters for bacterial genome were used.

Whole genome sequence reads were deposited in the NCBI SRA under BioProject ID PRJNA687382. The GenBank assembly accessions are GCA_018588045.1, GCA_018588075.1, GCA_018588055.1, GCA_018588025.1, GCA_018588125.1, GCA_018588105.1, GCA_018588135.1, GCA_018704025.1, GCA_018704075 and GCA_018587995.1

### 2.10. Typing

The typing of the whole genome sequenced strains *P. dorei* PtFD1P5, PtFD8M1, PtFD16P1, PtFD16M14, PtFSb6, PtFSb8, PtFC1P2, *B. uniformis* PtFD3Pch2, PtFSb3P5 and *P. distasonis* PtFD14MH1 was performed by using the multilocus sequencing typing (MLST) using 30 genes listed in Table S3 following the recommendations of Chun et al. and Munoz et al. [40,41]. The selected genes are involved in the housekeeping apparatus and the central metabolic pathways of the bacterial cell. The sequence alignment was performed using Clustal Omega [42], and the phylogenetic analysis and tree construction (using concatenated aligned sequences), was conducted with the software MEGA X [43]. As reference, the sequences of the listed genes of the strains *P. dorei* DSM 17855, CL03T12C01, CL02T12C06, CL02T00C15; HS1 L3 B 079, HS2 L2 B045b, HS1 L1B010, *B. uniformis* ATCC 8492 and *P. distasonis* ATCC 8503 were used.

### 2.11. Prophage and CRISPR-Cas Systems Detection

The *P. dorei*, *B. uniformis* and *P. distasonis* genomes were analyzed with PHASTER (PHAge Search Tool Enhanced Release) (http://phaster.ca/, accessed on 4 June 2021) [44] to search for potential prophage sequences. CRISPRs (clustered Regularly Interspaced Short Palindromic Repeats) and Cas (CRISPR-associated) proteins were searched using the CRISPRCasFinder [45].

### 2.12. Identification of Resistance, Pathogenicity Potential and Molecular Mimicry

In order to identify acquired antimicrobial resistance genes, the software CARD/RGI was used [46]. The identification of the potential pathogenicity to human host was conducted using the software PathogenFinder 1.1 [47]. The molecular mimicry identification was conducted using BLASTP (version 2.2.26) through SEED Viewer (version 2.0) by searching the presence of the insulin B chain insB:9–23 peptide mimics (SHLVEALYLVCGERG) described in *P. distasonis* 33B, which was found in the Human Gut Microbiome [48], and the two microbial altered peptides ligands (MVWGPDPLYV and RQFGPDWIVA) of the natural preproinsulin (ALWGPDPAAA) reported in *Bacteroides fragilis*/*thetaiotaomicron* and *Clostridium asparagiforme*, respectively [49].

### 2.13. Cell Culture Assays

The human colonic cell line HT29-MTX-E12 (ECACC 12040401) (Sigma-Aldrich, European Collection of Authenticated Cell Cultures, Porton Down, Salisbury, UK) was routinely cultured in Dulbecco’s Modified Eagle Medium (DMEM, Sigma-Aldrich, St. Louis, MO, USA) supplemented with 10% (*v*/*v*) fetal bovine serum (FBS), 1% (*v*/*v*) non-essential amino acids, and antibiotics (Penicillin/Streptomycin (PEN-STREP), 5000 units/mL Penicillin and 5000 µg/mL Streptomycin) (Gibco, Carlsbad, CA, USA) (DMEM complete). Cultures were incubated at 37 °C in a humidified 5% (*v*/*v*) CO_2_ atmosphere and grown until 80–90% confluence before being sub-cultured and used between passages 52 to 60.

For adhesion, invasion, and evaluation of the damage to the tight junctions assays, the cell tissue culture was performed for 21 days post seeding to reach mature HT29-MTX-E12 cells. Cells were seeded in 24-well culture plates at concentration of 4.0 × 10^4^ cells per well. The culture medium was changed every two days for the 21 days of the differentiation period. In the last medium change no antibiotics were added to the medium.

#### 2.13.1. Adherence Assay

The evaluation of the adhesion ability of strains of *P. dorei*, *B. uniformis* and *P. distasonis* to HT29-MTX-E12 intestinal cells was performed as previously described by Gagnon et al. [50] with some modifications. The bacterial cultures were grown in BHI + H in anaerobic conditions during 24 h at 37 °C. The previous bacterial culture was used to inoculate at 10^7^ CFU/mL the HT29-MTX-E12 cells prepared as previously described. The tissue culture plates were then incubated at 37 °C under anaerobic conditions for 30 min. After this time interval the non-adherent bacteria were eliminated by washing with (PBS) (twice). Subsequently, the cell line with adherent bacteria was treated with 250 μL Trypsin-EDTA (Sigma-Aldrich, St. Louis, USA per well and incubated at 37 °C for 10 min. To inactivate the Trypsin-EDTA, DMEM supplemented with 10% (*v*/*v*) of FBS was added to each well. From this suspension, serial decimal dilutions were performed in PBS, and then each dilution was inoculated on BHI + H agar plates. The inoculated plates were incubated under anaerobic conditions at 37 °C for 48 h.

The adhesion results are expressed as the percentage of the number of adherent bacterial cells relative to the total number of bacteria used on the assay.

#### 2.13.2. Invasion Assay

The evaluation of the invasion ability of strains of *P. dorei*, *B. uniformis* and *P. distasonis* to HT29-MTX-E12 intestinal cells was performed as previously described by Gagnon et al. [50] with some modifications. Briefly, the bacterial cultures were grown in BHI + H in anaerobic conditions for 24 h at 37 °C. The bacteria were then inoculated at 10^7^ CFU/mL into the plates with the HT29-MTX-E12 cells prepared as previously described. The tissue cell plates were then incubated at 37 °C under anaerobic conditions for 4 h. The infected cell lines were washed with PBS (twice) and exposed to 250 μL DMEM medium containing 150 μg/mL of Gentamicin (Sigma-Aldrich, Germany) per well for 1 h at 37 °C to eliminate any non-invasive bacteria. A new washing step was carried out with PBS, and 250 μL Trypsin-EDTA was added to each well with further incubation at 37 °C for 10 min. The cell lines were then exposed to 0.1% (*v*/*v*) Triton X-100 (Merck, Darmstadt, Germany) for 10 min at 37 °C to permeabilize the cells allowing the release of invasive bacteria. From this suspension, serial decimal dilutions were prepared, and the bacterial counts were performed as described above.

The results of the invasion ability are expressed as the percentage of the number of invasive bacteria relative to the total bacteria used in the assay.

#### 2.13.3. Ability of Bacteroides Species to Impair Epithelial Integrity

The capacity of *P. dorei*, *B. uniformis* and *P. distasonis* strains to cause alterations to the epithelial integrity of the cell line HT29-MTX-E12 was evaluated as previously described in [50] with slight modifications. The cells were seeded in wells containing a sterile coverslip, Ø 13 and 1.5 mm thickness (VWR, Radnor, Philadelphia, PA, USA). Bacterial suspensions at a concentration of 10^7^ CFU/mL were transferred to the epithelial intestinal cells after the differentiation period of 21 days. The cell line was exposed to the bacterial cells during 4 h at 37 °C under anaerobic conditions. The infected cells were then washed with PBS 3 times and fixed with 3.7% (*v*/*v*) formaldehyde (Labscan Analytical Sciences, Gliwice, Poland) for 15 min. Three new washing steps with PBS were performed. The cell line was then permeabilized with 0.5% Triton X-100 (Merk, Darmstadt, Germany) for 10 min, followed by 3 washing steps with PBS. The cells were then blocked for non-specific binding with 3% (*w*/*v*) bovine serum albumin (Merk, Germany) at 4 °C for 1 h. Thereafter the cells were incubated during 20 min with tetramethylrhodamine B isothiocyanate-phalloidin (TRITC-phalloidin) (Sigma-Aldrich, Hamburg, Germany) (diluted 1:200 from an initial solution of 0.1 mg/mL). Afterwards the cell line was exposed for 3 min to 4′,6′-diamidine-2-phenylindole (DAPI) (Sigma-Aldrich, Germany) (diluted 1:200 of an initial solution of 14.3 mM). Two final washes were carried out with PBS. The assays were performed using three biological and two technical replicates.

Each coverslip was then mounted inverted on a microscope slide with a drop of Fluromount (Sigma-Aldrich, Germany). The observation of the cell lines was performed using the microscope Axio Imager Z2 (Zeiss, Oberkochen, Germany).

#### 2.13.4. Exposure of Intestinal Epithelial Cells to the Bacteroides Metabolome (Secretome)

The evaluation of the effect of the *Bacteroides* metabolome on the viability of the cell line HT29-MTX-E12 was performed using the Vibrant MTT cell proliferation assay kit (Molecular Probes, Invitrogen, Eugene, OR, USA) following the manufacturer instructions. Briefly, the bacterial cells were grown in BHI + H at 37 °C for 24 h under anaerobic conditions. Thereafter the bacterial suspensions were centrifuged (5000× *g*, 10 min, 4 °C) and the supernatant was collected and filtered (0.2 µm pore diameter, Whatman, GE Healthcare, Buckinghamshire, UK). The supernatant samples were stored at −20 °C until use.

HT29-MTX-E12 cells required were seeded in a 96-well microplate (8000 cells per well) and incubated for 48 h at 37 °C in a 5% (*v*/*v*) CO_2_ humidified atmosphere. Afterwards DMEM complete was discarded and replaced with DMEM complete supplemented with the bacterial supernatant at different percentages (10, 25 and 50% [*v*/*v*]). Three biological and 4 technical replicates were used. The pH value of the different combinations was 7.8 ± 0.11 for the 10% (*v*/*v*) mix, 7.53 ± 0.12 for 25% (*v*/*v*) and for 50% (*v*/*v*) the pH was 7.21 ± 0.26. The cell line was exposed to the bacterial supernatant for 24 h. After this time interval, the DMEM medium with the supernatant was replaced with DMEM complete, and the MTT solution was added to each well. The cell line was then incubated at 37 °C in a 5% (*v*/*v*) CO_2_ humidified atmosphere for 4 h. Thereafter an SDS-HCl solution was gently added into each well followed by an incubation for 4 h at 37 °C in a 5% (*v*/*v*) CO_2_ humidified atmosphere. Finally, each well was homogenized to eliminate any precipitate and the absorbance value was determined at 570 nm using a microplate reader (Tecan Infinite, M200, Männedorf, Switzerland).

#### 2.13.5. Invasion Ability of Bacteroides Species in the Presence of *Lactobacillus casei*

The ability of strains of *P. dorei*, *B. uniformis* and *P. distasonis* in the presence of *Lactobacillus casei* DSM 20011 to invade HT29-MTX-E12 intestinal cells was performed as described above. The *L. casei* DSM 20011 was grown on Man, Rogosa and Sharpe (MRS) medium under microaerophilic conditions for 24 h at 37 °C. *Bacteroides* and *L. casei* were inoculated at 10^7^ CFU/mL to the intestinal epithelial cells. The incubation conditions and recovery of bacterial cells that were able to invade were performed as described in Section 2.13.2*,* except that *L. casei* cells counts were conducted using the MRS medium under microaerophilic conditions, whereas *Bacteroides* spp. were performed in BHI + H supplemented with vancomycin (15 μg/mL) and kanamycin (200 μg/mL) and the incubation was conducted in anaerobic conditions.

The results are expressed as the percentage of the number of invasive bacteria relative to the total bacteria used in the assay.

### 2.14. Statistical Analysis

Data was analyzed for statistical significance by one or two-way ANOVA with the software SPSS (version 25) (Inc., Chicago, IL, USA). Statistical significance was considered at *p* < 0.05; when the analysis was statistically significant, the Tukey’s post hoc test was performed. Violin plots on bacterial abundances with qPCR results were performed using GraphPad Prism (version 9.0) (GraphPad Software, San Diego, CA, USA).

## 3. Results

### 3.1. Counts and Diversity of Bacteroides Species

The number of *Bacteroides* species recovered from culture achieved similar values (*p* > 0.05) between T1D and control samples; 6.64 ± 0.74 Log_10_ CFU/g feces for T1D children and 6.40 ± 0.06 Log_10_ CFU/g feces for control children.

**Figure 1:**
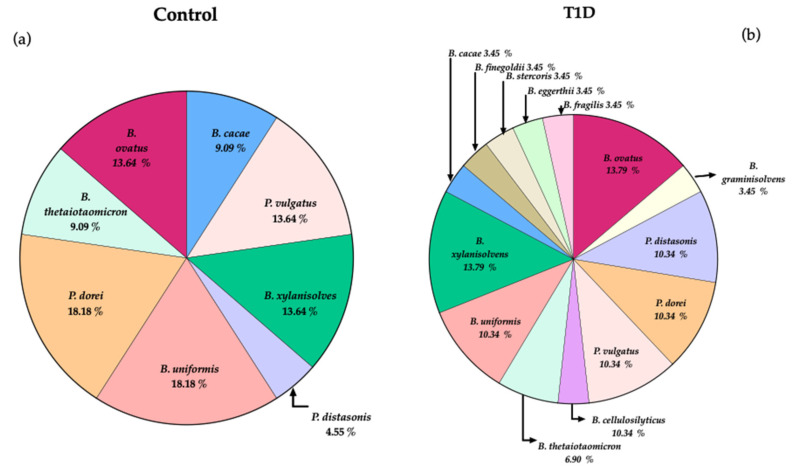
*Bacteroides* species recovered in culture from (**a**) control and (**b**) T1D fecal samples.

The different *Bacteroides* species isolated from the fecal samples of the two groups are illustrated in Figure 1. Several *Bacteroides* species were only isolated from T1D samples, namely the species *B. graminisolvens*, *B. cellulosilyticus*, *B. finegoldii*, *B. stercoris*, *B. eggerthii* and *B. fragilis* that achieved 3.45% each and none from controls. The recovery of *P. dorei* in culture was attained from only three T1D samples reaching 10.34%, whereas in controls *P. dorei* was recovered from four samples achieving 18.18%. In T1D samples several *Bacteroides* species were recovered at similar percentages to *P. dorei*, such as *B. uniformis* and *P. vulgatus* (10.34%). The percentage of recovery of *B. ovatus* and *B. xylanisolvens* was similar for both groups (13.79%).

Overall, the core of *Bacteroides* species recovered from the two groups, besides *P. dorei*, includes the species *B. ovatus*, *P. vulgatus*, *B. uniformis* and *B. xylanisolvens*. Interestingly the recovery of *P. distasonis* was higher from T1D samples (10.34%) in comparison with control samples (4.55%).

**Figure 2:**
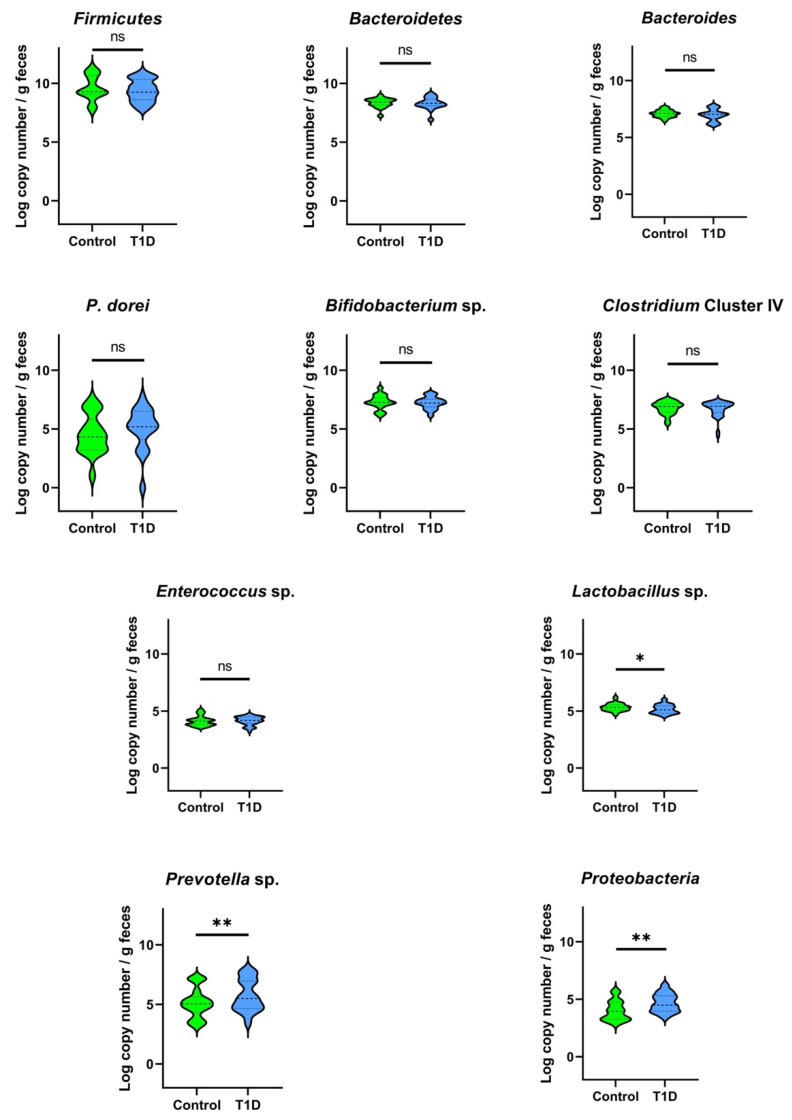
Violin plots reporting the qPCR quantification of fecal bacterial groups in T1D and control children (*n* = 17 participants per group). *p*-values were computed using the Mann–Whitney U test, * *p* < 0.05, ** *p* < 0.001, ns—not significant.

The load of the targeted bacterial groups in the fecal samples of T1D and control children determined by qPCR is illustrated in Figure 2. Similar amounts (*p* > 0.05, and values presented as Log_10_ copy number/g feces) for both groups were found for *Firmicutes* (control 9.63 ± 1.09, T1D 9.39 ± 0.93), *Bacteroidetes* (control 8.35 ± 0.40, T1D 8.31 ± 0.51), *Bacteroides* sp. (control 7.10 ± 0.32, T1D 6.97 ± 0.52), *P. dorei* (control 3.97 ± 1.52, T1D 4.21 ± 1.43), *Bifidobacterium* sp. (control 7.30 ± 0.60, T1D 7.22 ± 0.51), *Clostridium* cluster IV (control 6.80 ± 0.55, T1D 6.70 ± 0.68), and *Enterococcus* sp. (control 4.14 ± 0.43, T1D 4.15 ± 0.38). However, T1D children carried lower numbers of *Lactobacillus* sp. (*p* < 0.05) in comparison with control children, but a higher abundance (*p* < 0.05) of *Prevotella* sp. and *Proteobacteria* was observed in their fecal samples.

### 3.2. Antibiotic Susceptibility

*Bacteroides* species are the most implicated anaerobic bacteria in infections, and resistance to antibiotics varies among the different species and also across geographic regions [31,51]. To evaluate the antibiotic susceptibility, a set of *Bacteroides* isolates was selected and their susceptibility was determined by disc diffusion including the antibiotics amoxycillin–clavulanate, clindamycin, imipenem, moxifloxacin, piperacillin-tazobactam, and metronidazole. The tested antibiotics are used in the treatment of infections caused by anaerobic bacteria [31,32,33,51]. The results of the antibiotic susceptibility are summarized in Table S4. Regarding the susceptibility of *P. dorei* strains for the antibiotic amoxycillin–clavulanate, two resistant strains in T1D group were observed and in the control group three resistant strains were detected. More than half of the *P. dorei* strains were resistant to clindamycin (58%), and even in the control group three strains were resistant to this antibiotic. For imipenem about 92% of the *P. dorei* strains were resistant, including the strains from the control group where just 1 strain was susceptible to IMP. In contrast 83% of the *P. dorei* strains were susceptible to moxifloxacin, and it was on the control group that it was found two resistant strains. For piperacillin–tazobactam more than half of the strains were resistant (58%) and they were observed in both groups. The strains of *B. uniformis* and the isolate of *P. distasonis* were susceptible to amoxycillin–clavulanate, and just two strains were susceptible to clindamycin. For imipinem only two strains of *B. uniformis* were susceptible. In contrast just one strain of *B. uniformis* was resistant to moxifloxacin belonging to the control group. The resistance to piperacillin–tazobactam was detected in one strain of *B. uniformis* isolated from control children (*B. uniformis* PtFC3M3). All the strains of *P. dorei*, *B. uniformis* and *P. distasonis* were susceptible to metronidazole (Table S4).

The epsilon test for the antibiotic imipinem evidenced that all tested *P. dorei* strains were susceptible to this antibiotic, in contrast with the disc diffusion assay for which only one *P. dorei* strain was susceptible (Table S5). The strain of *B. uniformis* PtF Sb3P5 and *P. distasonis* PtFD14MH1 that according to the disc diffusion test were resistant, using the epsilon these strains are susceptible. The epsilon test for MTZ (0.016–256 µg/mL) evidenced MIC values between 0.0195–0.250 µg/mL (breakpoint S ≤ 4 R ≥ 4 mg/L) confirming the disc diffusion results; all tested strains of *P. dorei*, *B. uniformis*, and *P. distasonis* PtFD14MH1 were susceptible to this antibiotic.

The results of the *in silico* analysis of the genome of the strains *P. dorei* PtF D1P5, PtF D8M1, PtF D16P1, PtF D16M14, PtF Sb6, PtF Sb8, PtFC1P2, *B. uniformis* PtF D3Pch2, PtF Sb3P5 and *P. distasonis* PtF D14MH1 for the identification of resistance genes using the CARD/RGI are summarized in Table S6. The *adeF* gene that belongs to the antimicrobial resistance (AMR) gene family of resistance-nodulation cell division (RND) antibiotic efflux, and confers resistance to fluoroquinolone and tetracycline antibiotics, was detected on all strains, and in some strains more than one copy was present. Interestingly, the strains *P. dorei* PtF D16P1 and PtF D16M14 only carry this resistance gene.

The *mef* (En2) gene that belongs to the AMR gene familiy of major facilitator superfamily (MFS) antibiotic efflux pump that confers resistance to macrolide antibiotic was detected in four strains of *P. dorei*; PtF D1P5, PtF D8M1, PtF Sb6 and PtF Sb8. The tetQ gene that belongs to the AMR gene family tetracycline-resistant ribosomal protection protein was identified in six strains; five strains of *P. dorei* (PtF D1P5, PtF D8M1,PtF Sb6 PtF Sb8, PtF C1P2) and in the *B. uniformis* strain PtF D3Pch2. The gene *ErmF* that belongs to the Erm 23S ribosomal RNA methyltransferase family was detected in two strains of *P. dorei*, PtF D8M1 and PtF Sb8 and in the *B. uniformis* strain PtF Sb3P5. *P. distasonis* PtF D14MH1 carries a gene that encodes a beta-lactamase of the CfxA family, CfxA2 that has been found in *Prevotella intermedia* [52]. The strain of *B. uniformis* PtF Sb3P5 carries two beta-lactamase genes, namely OXA-347and CblA-1 that is found in *B. uniformis* and is species specific [53,54]. Single nucleotide polymorphisms (SNPs) were not detected.

### 3.3. Mucinolytic Activity

In defined medium with glucose as sole carbon source, all *P. dorei* strains and the strain of *P. vulgatus* PtFD2P1 reached a four fold growth in this medium. In contrast, the fold change in the defined medium with PGM was only one fold for all tested strains (Figure S1). The growth in the rich medium BHI attained a two fold increase, and the strain of *P. dorei* PtF D1P5 was the one that reached slighty higher than two fold in BHI (Figure S1).

Mucin degradation was revealed by the amidoblack staining of solidified defined medium with PGM where the cultures of the the strains of *P. dorei* and *P. vulgatus* caused the appearance of clear zones demonstrating mucinolytic activity. The cultures of *E. coli* strain D3 and C1 showed no mucin degradation zones around their growth evidencing their inability to degrade mucin (data not shown).

### 3.4. Repetitive Based Sequence PCR 

The PCR patterns generated either with BOX primer (BOX-PCR) or the ERIC primers (ERIC-PCR) distinguished the different *Bacteroides* species, *P. dorei*, *P. vulgatus* and *P. distasonis* isolates (Figure S2). The cluster analysis of BOX-PCR fingerprints grouped the *P. dorei* isolates in three groups; the reference strain *P. dorei* DSM 17855 grouped with *P. dorei* PtFSb8 (80.8% similarity) and the second cluster included the main isolates (PtFD16P1, PtFD16M14, PtFSb19Pp7, PtFSb6, PtFD1P5, PtFD1P20, PtFD1P21 and PtFD8M1). Two *P. dorei* isolates (PtFC1P2 and PtFC1M8) grouped with *P. vulgatus* (73.4% similarity).

The cluster analysis based on the BOX-PCR for *B. uniformis* identified two main clusters; the first included the isolates *B. uniformis* PtFD3P28, PtFSb3P5 and Sb13P5 (97.3 and 96.6%) and the second included the isolate *B. uniformis* PtFD6P31with 87.6% similarity between them. The isolate *B. uniformis* PtFC7NB15 showed low relatedness with the two main clusters (33.1%). Two other isolates of *B. uniformis* (PtFC3M3 and PtFC6Pp6) clustered with *B. thetaiotaomicron* with 66.9% similarity. Interestingly, the isolate *B. uniformis* D3Pch2 showed a singular singleton clustering with *B. ovatus* (59.1%) (Figure S2A).

Analysis of banding patterns produced using the BOX rep-PCR for *B. xylanisolvens* formed two main clusters, one includes the isolates from a unique T1D child, PtFD6Phid, PtFD6M1, PtFD6M10, PtFD6P1, PtFD6P5 achieving 99% similarity, indicating that they can constitute a single strain. This grouping for these *B. xylanisolvens* isolates was also observed using the ERIC rep-PCR (Figure S3). The second cluster of *B. xylanisolvens* includes the isolates PtFD4M1, PtFD13MH1, PtFD3P4, PtFD3M6, PtFD3P6, PtFSb3P12, PtFSb3M1 showing high similarity (73.3%). Other *B. xylanisolvens* isolates clustered with other *Bacteroides* species, namely the isolate *B. xylanisolvens* PtFD4P14 clustered with two isolates of *B. ovatus* with 93.2% relatedness, *B. xylanisolvens* PtFSb9MH2 clustered with the isolate of *B. fragilis* PtFD13MH3 with 98.1% similarity and the isolate *B. xylanisolvens* PtFSb2Phid clustered with *P. vulgatus* PtF D17P19 showing 78.1% similarity.

The BOX rep-PCR banding pattern analysis for *B. thetaiotaomicron* grouped the isolates PtF C6P14, PtFC6P14Smo achieving 97.9% similarity and the isolate PtFD4P1 was related with this cluster with 88% similarity. Two other *B. thetaiotaomicron* isolates PtFD4M3 and PtFD4 M11 (92.2%) were related with the other isolates at 85.1% similarity. The isolate *B. thetaiotaomicron* PtFD2P14 was a singleton showing 61.0% similarity with the other isolates. Two other *B. thetaiotaomicron* singletons were identified, the isolate PtFD4P11 and the isolate PtFC2Phid (Figure S2A).

The cluster analysis of ERIC-PCR fingerprints, as stated above, discriminated the *Bacteroides* isolates by species (Figure S2B). The *P. dorei* isolates were grouped differently from the BOX-PCR producing three groups (Figure S2B). The first group includes the isolates *P. dorei* PtFC1M8, PtFD1P20, PtFD1P20, PtFSb8, the second includes the isolates PtFD16P1 and PtFD16M14, and the third group includes the isolates PtFD1P5 and PtFSb6. The *P. dorei* PtFC1P2 forms a singleton with a very low number of bands produced by ERIC primers, evidencing 71.9% similarity with *B. stercoris* and *B. finegoldii* (Figure S2B). A very lower number of bands produced by ERIC-PCR primers is observed in two *B. uniformis* isolates; PtFC3M3 and PtFC7NB15. Singletons in ERIC-PCR cluster analysis were identified in several *Bacteroides* species, namely *B. thetaiotaomicron* PtFD2P14, *B. uniformis* PtFD3Pch2 and PtFC6Pp6, *B. xylanisolvens* PtFD4P14 and PtFD4M1, *B. ovatus* PtFC4P5 and PtFC5M11 (Figure S2B).

### 3.5. Interaction with Intestinal Epithelial Cells

**Figure 3:**
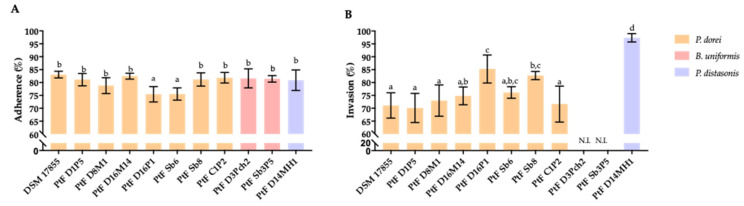
(**A**) Adherence and (**B**) invasion ability (%) of the *Bacteroides* species tested with HT29-MTX-E12 cell line. Bars with the same lowercase letter are not statistically different (*p* > 0.05). N.I.—non-invasion.

The interaction of *P. dorei* strains with intestinal epithelial cells was evaluated using the cell line HT-29-MTX-E12. The ability of *P. dorei*, *B. uniformis* and *P. distasonis* to adhere and invade the HT-29-MTX cells is illustrated in Figure 3. All tested strains of *P. dorei*, *B. uniformis* and the strain of *P. distasonis* PtFD14MH1 were equally (*p* > 0.05) able to adhere to HT-29-MTX cells, except the *P. dorei* strain PtFD16P1 and PtF Sb8 that showed a lower adherence (*p* < 0.05) (Figure 3A). The highest ability to invade was displayed by the strain of *P. distasonis* PtFD14MH1 (*p* < 0.05). The invasion ability of *P. dorei* PtF D16P1 and PtF Sb8 was higher (*p* < 0.05) in comparison with the other *P. dorei* strains. The *B. uniformis* strains PtF D3Pch2 and PtF Sb3P5 were not able to invade HT-29-MTX cells (Figure 3B).

**Figure 4:**
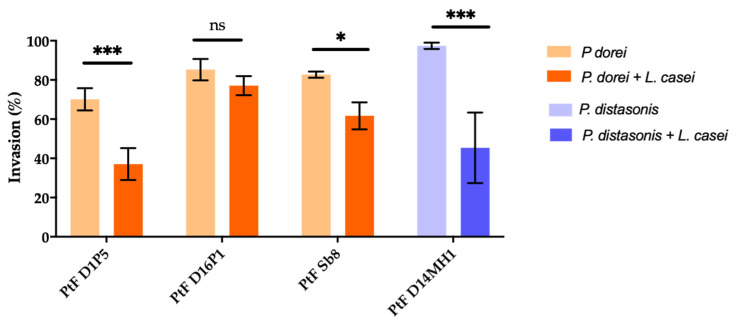
Effect of the presence of *Lactobacillus casei* DSM 20011 on the invasion ability (%) of *P. dorei* strains (*P. dorei* + *L. casei*) and *P. distasonis* (*P. distasonis* + *L. casei*). * *p* < 0.05; *** *p* < 0.001, ns—not significant.

Since T1D children showed a lower number of *Lactobacillus* sp. by qPCR (Section 3.1) the impact of *L. casei* DSM 20011 on the invasion ability of *P. dorei* strains PtF D1P5, PTF D16P1, PtFSb8 and *P. distasonis* D14MH1 was evaluated. The results are illustrated in Figure 4. The invasion ability of the *P. dorei* strains was significantly diminished in the presence of *L. casei* (*p* < 0.05–*p* < 0.01), except for the strain *P. dorei* PtF D16P1, whose invasion ability was not affected by the presence of *L. casei* (*p* > 0.05) (Figure 4).

#### 3.5.1. The Viability of HT29-MTX-E12 Cells Exposed to the Secretome of Bacteroides Species

**Figure 5:**
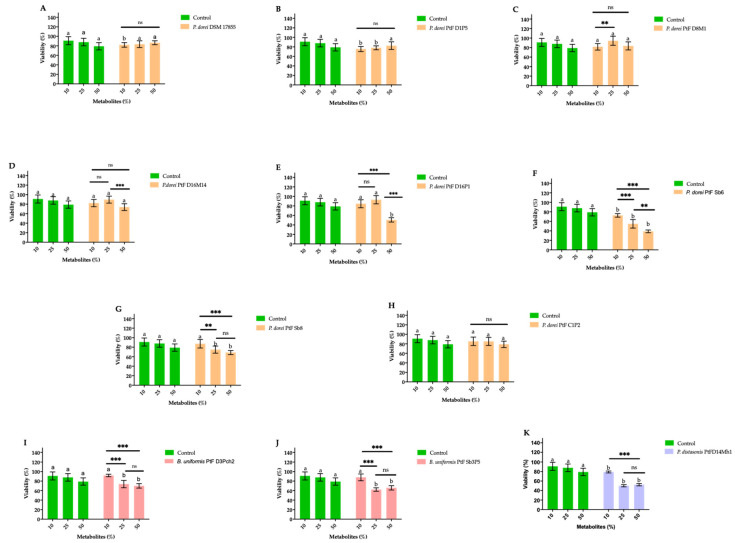
Viability of the HT29-MTX-E12 cells exposed to 10%, 25% and 50% of the secretome of the culture of *P. dorei* DSM 17855 (**A**), *P. dorei* PtFD1P5 (**B**), *P. dorei* PtFD8M1 (**C**), *P. dorei* PtFD16M14 (**D**), *P. dorei* PtFD16P1 (**E**), *P. dorei* PtFSb6 (**F**), *P. dorei* PtFSb8 (**G**), *P. dorei* PtFC1P2 (**H**), *B. uniformis* PtFD3Pch2 (**I**), *B. uniformis* PtFSb3P5 (**J**), *P. distasonis* PtFD14MH1 (**K**). The addition of BHI to cell culture at the tested concentrations was used as control. Bars with the same letter are not statistically different when compared to control (*p* > 0.05). Data statistically different for each strain are represented by ** *p* < 0.01; *** *p* < 0.001.

In order to evaluate the potential impact of the secretome produced by the *Bacteroides* species on the viability of intestinal epithelial cells, HT-29-MTX-E12 cells were exposed for 24 h to the culture medium supplemented with different percentages (10%, 25% and 50%) of the secretome of *Bacteroides* cultures. The results are illustrated in Figure 5.

The increase of the secretome concentrations of the reference strain *P. dorei* DSM 17855, *P. dorei* PtFD1P5, *P. dorei* PtFD8M1, and *P. dorei* PtFC1P2 did not significantly impaired (*p* > 0.05) the viability of HT-29-MTX-E12 cells. In contrast, the exposure of HT-29-MTX-E12 cells to increasing concentrations of the bacterial culture secretome of the strains *P. dorei* PtFD16M14, *P. dorei* PtFD16P1, *P. dorei* PtFSb6, *P. dorei* PtFSb8, *B. uniformis* PtF D3Pch2, *B. uniformis* PtF Sb5P5, and *P. distasonis* PtFD14MH1 significantly affected the viability of the intestinal epithelial cells (*p* < 0.05) (Figure 5).

#### 3.5.2. Integrity of HT29-MTX-E12 Cells after Exposure to Bacteroides Species

The different strains of *P. dorei* were able to adhere and invade the HT-29-MTX-E12 cells, except the two tested strains of *B. uniformis* that were not able to invade. Therefore, the impact of colonization by different bacterial species on tight junction integrity was investigated. The HT-29-MTX-E12 cells were stained with TRICT-phalloidin, a phallotoxin that binds to actin filaments. This binding allows the observation of any damage that has occurred in the network of tight junctions as a result of the injurious action of the *Bacteroides* species compromising the normal functioning of paracellular communication.

**Figure 6:**
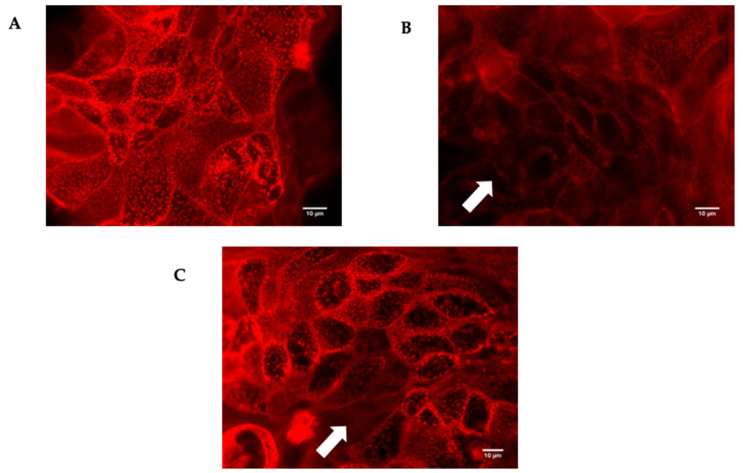
TRITC-phalloidin staining (red) of cell junction’s of the cell line HT29-MTXE12. No colonized cells (**A**), and after 4 h of colonization with *P. dorei* PtF D16P1 (**B**) and *B. uniformis* PtF D3Pch2 (**C**). The white arrow indicates evidence of the cell junction’s disruptions after the contact with *P. dorei* PtF D16P1 and *B. uniformis* PtF D3Pch2 in comparison with the undisrupted cell junctions of the control monolayers.

The damage to the tight junctions of HT29-MTX-E12 cells after exposure to *P. dorei* strains and *P. distasonis* was similar and considerable, in contrast with the cells exposed to *B. uniformis* that were only slightly affected. A representative image of the disturbed tight junctions by the colonization of HT29-MTX-E12 cells with the strain *P. dorei* PtF D16P1 and *B. uniformis* PtF D3Pch2 is illustrated in Figure 6.

### 3.6. Genomic Features

**Figure 7:**
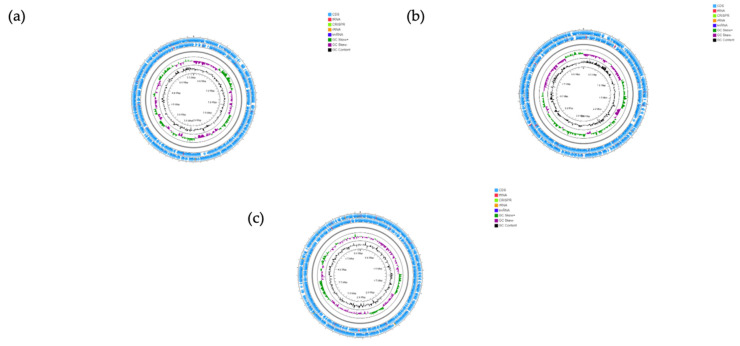
Genome map of *P. dorei* PtFD16P1 (**a**), *B. uniformis* PtFD3Pch2 (**b**), and *P. distasonis* PtFD14MH1 (**c**). The outermost ring depicts the coding sequences in both clockwise and anti-clockwise direction. tRNA is depicted as red arrow heads, rRNA in orange and tmRNA in royal blue. CRISPR repeats regions are coloured in lime green. GC skewing is shown in green and purple, and GC content in black.

Representative circular genome maps for *P. dorei*, PtFD16P1, *B. uniformis* PtFD3Pch2 and *P. distasonis* PtFD14MH1 is shown in Figure 7 (the genome map of the remaining seven strains is presented in Figure S3), and their main features are listed in Table 1. The genome size of *P. dorei* strains ranged from 5120 Mb to 5880 Mb, and the genome size of *P. distasonis* PtFD14MH1 (5066 Mb) is similar to the *P. dorei* strains (Table 1). The genomes of *P. dorei* strains were distributed along 62 to 153 contigs, with the number of contigs for the strain of *P. distasonis* PtFD14MH1 being 81. *B. uniformis* PtFD3Pch2 showed a similar genome size (5457 Mb) to *P. dorei* strains whereas *B. uniformis* PtF Sb3P5 showed the lowest genome size, 4393 Mb, assembled in 8 and 21 contigs, respectively. The observed GC% content for the *P. dorei* strains ranged between 41.4% and 42.2%. In contrast *B. uniformis* strains and *P. distasonis* showed the highest GC % content, *B. uniformis* PtFD3Pch2 achieves 44.1%, *B. uniformis* PtF Sb3P5 46.4%, and 45% for *P. distasonis* PtFD14MH1.

The number of coding genes varied among the *P. dorei* strains, namely the highest number was displayed by the strain PtFD1P5 (5184) and PtFSb6, and the lowest by the strain PtFSb8 (4288) followed by the strain PtFD8M1. The number of coding genes in *P. distasonis* PtFD14MH1 was similar to those of *P. dorei* strains (4240). The strains of *B. uniformis* PtFD3Pch2 and PtF Sb5P5 showed 4597 and 3644 coding sequences, respectively.

The distribution of genes by functional categories produced by RAST is summarized in Table S7. Overall, the number of genes in each functional category did not vary markedly between the strains. Some of the categories are highlighted. In the category Phages, Prophages, Transposable elements, and Plasmids, the number of genes in the genomes of *P. dorei* PtFD8M1, PtFSb8, and *P. distasonis* PtFD14MH1 are 47, 48, and 33 respectively. However, in this category it is found in the genomes of *P. dorei* PtFD16P1 and PtFSb8, 4 copies of a thiol-activated cytolysin (LLO) of the subsystem of *Listeria* Pathogenicity Island LIPI-1. Three copies of this same LLO gene are present in the genomes of strains *P. dorei* PtFD16M14, PtFD8M1, and PtFC1P2. However, no copies of the thiol activated cytolysin were detected in the genome of *P. dorei* PtFSb6. The same four copies number of the thiol-activated cytolysin was found in the genome of *B. uniformis* PtFD3Pch2. In contrast, in the genome of *B. uniformis* PtFSb3P5 just 1 copy of the phosphatidylinositol-specific phospholipase C (PlcA) was identified. In the genome of *P. distasonis* PtFD14MH1, two copies of this type of the thiol-activated cytolysin were identified. The percentage identity of the identified thiol-activated cytolysins with the listeriolysin O of *L. monocytogenes* EGD (lmo0202) is 42.19% for the strain *P. dorei* PtF D16P1 (20547_PtFD16P1_01393), 39.98% for *B. uniformis* PtFD3Pch2 (20551_D3Pch2_00006) and 48.50% for *P. distasonis* D14MH1.

The dendogram obtained from the multilocus sequencing typing is composed of 8 clusters in total (Figure S4). *P. dorei* strains are distributed in 6 clusters, and the strains of *B. uniformis* and *P. distasonis* are clustered in two independent clusters. The *P. dorei* strains clustered differently with the *P. dorei* strains used as reference. The reference strain *P. dorei* DSM 17855 is the one that is more distant.

The identification of the potential pathogenicity for the human host, performed with PathogenFinder 1.1, revealed the presence of a collagenase precursor (ABR38775) in the *P. dorei* strains PtF D1P5, PtFD16P1, PtFD16M14, PtFC1P2, and *B. uniformis* PtFD3Pch2. In *P. dorei* PtFD16P1 a putative membrane peptidase (ABR39331) was detected. Interestingly, in the genome of *P. distasonis* PtFD14MH1 it was found 4 hemolysin related proteins (ABR 43727,ABR 42607, ABR 44661, and ABR 42685), a metal-dependent hydrolase (ABR42910), a putative hydrolase (ABR 42975), a *N*-acetyl-muramoyl-l-alanine-amidase (ABR 44579), a putative alkaline protease AprF (ABR 41930), a putative aminopeptidase (ABR 42007), and a putative capsule polysaccharide export protein (ABR42909).

Prophage sequences identified by PHASTER are summarized in Table S8. Only incomplete prophages were identified. Among the analyzed bacterial genomes, *Flavobacterium* phages were the most common putative phage identified, except for *P. dorei* PtFD1P5, and *P. distasonis* PtFD14MH1. Interestingly, the strain of *P. dorei* PtF C1P2 carry a prophage DNA adenine methylase enzyme (Dam) (PHAGE_Flavob_vB_FspS_morran9_1_NC_048836), and the strains PtFD8M1 and PtFSb8 also show a *Flavobacterium* phage DNA adenine methylase enzyme (PHAGE_Flavob_vB_FspM_pippi8_1_NC_048830).

*B. uniformis* PtFD3Pch2 and PtF Sb3P5 show an endolysin from a *Rhodococcus* phage (PHAGE_Rhodoc_Trina_NC_042040), and a holin from a *Bacillus* phage (PHAGE_Bacill_Pookie_NC_027394).

Different sequences of CRISPR were detected in the analyzed genomes (Table S9). Three main CRISPR-Cas types, corresponding essentially to Type IC, Type IIC and Type VIA were identified.

Table 2 summarizes the results of searching for molecular mimicry in the analyzed genomes to identify the presence of insulin B chain insB:9–23 peptide mimics (SHLVEALYLVCGERG) [48], and the two microbial altered peptide ligands (MVWGPDPLYV and RQFGPDWIVA) from the natural preproinsulin (ALWGPDPAAA) [50]. A variant of the insB:9–23 peptide (HL**L**EALY**MTY**GE) was identified only in the genome of *B. uniformis* PtF Sb3P5. In contrast, in all *P. dorei* genomes and in the genome of the strain *B. uniformis* PtFD3Pch2, a modified version of the altered peptide MVWGPDPLYV (**MV**WGPD**NFYV**) is observed. Interestingly, the strain of *B. uniformis* PtFD3Pch2 showed another variant of this peptide (**MV**W**S**PDP**LYV**). The *P. distasonis* PtFD14MH1 showed an altered version of the peptide ligand RQFGPDWIVA.

## 4. Discussion

Nowadays it is clear that geographical location strongly influences the incidence of T1D [8,10,12,23]. Even in neighboring countries, the intestinal bacterial patterns observed in children with established T1D does not overlap [17,18,20]. This study confirms that Portuguese (Algarve region) children with T1D show similar *Bacteroides* counts, when compared with control children, both by culture-dependent and qPCR approachs. As it can be anticipated, the slight difference observed between the counts of *Bacteroides* by the culture-dependent approach and qPCR, is clearly associated with the lower recovery of bacteria in culture medium. Different *Bacteroides* species were recovered only from T1D children, namely *B. graminisolvens*, *B. cellulosilyticus*, *B. finegoldii*, *B. stercoris*, *B. eggerthii* and *B. fragilis*. Diet can modify the composition of the gut microbiota [54,55,56,57]. *Bacteroides* species are particularly modulated by the accessibility and abundance of dietary fiber [55,58,59], for example arabinoxylans that are naturally found in all major cereal grains increases the abundance of *B. cellulosilyticus* but not of *B. ovatus* [55,60]. The recommendations in the guidelines for nutritional management in children and adolescents with diabetes, stress the importance of consuming a variety of healthy foods, including fruits, vegetables, dairy, whole grains, and legumes [61]. However, the individual consumption of these healthy foods (with a significant load on dietary fiber) will impact the presence of different *Bacteroides* species in the gut of T1D children [54,55].

Another interesting finding was the higher recovery in culture of *P. distasonis* isolates from the fecal samples of T1D children in comparison with control children. Interestingly, Falony et al. [62] found an increase in *Parabacteroides* in individuals with low microbiome diversity that exhibited a preference for white, low fiber bread, and predominance of recent amoxicillin treatment. The recent reported molecular mimics of the human insulin B:9–23 peptide in *P. distasonis* 33B (from the human gut microbiome) [48] is an important warning for hidden autoimmune triggers. The genome of *P. distasonis* PtFD14MH1 did not show the presence of any variant of insB:9–23, however a variant of the Human Natural preproinsulin AWL, that is very similar to a previously described variant in C. *asparagiforme,* was identified [49]. The role of *P. distasonis* in host health is still controversial, namely while Huang et al. [48] reported the stimulation by *P. distasonis* 33B of the human T cell clones specific to insB:9–23 and the colonization of the gut of female NOD mice with *P. distasonis* 33B stimulate T1D onset, Wang et al. [63] reported that the treatment of obese mice with live *P. distasonis* resulted in an increase in the concentration of succinate in the jejunum, together with the activation of intestinal gluconeogenesis, diminishing food intake, and amelioration of glucose homeostasis. Other beneficial features of *P. distasonis,* is its ability to mitigate obesity-driven colorectal tumorigenesis and attenuate intestinal inflammation [64,65]. Altogether these findings reveal the controversial nature of the role of *P. distasonis* on gut inflammatory diseases. Nevertheless, it is reasonable to anticipate that such contrasting behavior may be highly strain-specific, and obviously the genetic background of the host is also a decisive factor. The reason for the higher recovery of *P. distasonis* isolates from T1D in comparison to control samples, and how their molecular mimicry can contribute to the development of T1D, requires additional research.

Repetitive sequence-based PCR banding patterns obtained from both BOX and ERIC primers, grouped the different *Bacteroides* species, but no discrimination by origin (cases and control) was observed. The rep-PCR method is directed to DNA sequences that are dispersed in the genome of the microorganism, and have been used to find differences in the bacterial genomes that will help with the discrimination between bacterial isolates, including the different *Bacteroides* species [30]. As the majority of bacteria, *Bacteroides* carry mobile genetic elements, such as plasmids, conjugative and mobilizable transposons [66]. In the host, these mobilizable genetic elements can enable the exchange of external DNA from the gut members, which can result in different rep-PCR banding profiles. The lack of differentiation of the *Bacteroides* species according to their sampling origin, lead us to assume that the host condition (T1D or healthy) has no impact on the coevolution of these *Bacteroides* species.

T1D children showed a similar ratio of *Firmicutes* to *Bacteroidetes* when compared to control children, but a significant increase in *Proteobacteria* and *Prevotella* sp., which is in contrast with the findings of Murri et al. [17] that observed a significantly higher ratio of *Firmicutes* to *Bacteroidetes,* and higher numbers of *Prevotella* sp. in healthy children with no significant differences in the numbers of *Proteobacteria*. The deviation of data regarding these bacterial groups can be justified by differences in geographical location factors [10,12,23]. However, the number of *Lactobacillus* sp. was impaired in T1D children, which is in line with the results reported by Murri et al. [17] in children with established T1D, and with the findings described by Biassoni et al. [67] in children at T1D onset, as well as with findings using rodent models, namely in bio-breeding diabetes prone mice [68] and streptozotocin T1D rat model [69,70].

All tested *P. dorei* and *P. vulgatus* strains were able to grow in the presence of porcine gastric mucin (1% [*w*/*v*]), but in a limited way when compared with their growth in the presence of glucose (0.5%, *w*/*v*) or in complex medium (BHI + H). The ability to break mucin, evidenced by amidoblack staining, was confirmed for all tested strains. *Bacteroides* species are recognized by their ability to degrade dietary fiber and host intestinal mucin [57,71]. Taking this into account, and knowing that hyperglycemia affects the intestinal mucus layer allowing a diminished microbiota-epithelial distance [72], *P. dorei*, *P. vulgatus* and other *Bacteroidetes* members in T1D gut may contribute to a disturbed mucus layer, therefore facilitating bacterial infiltration.

The ability to adhere to HT-29-MTX cells was similarly observed for all tested strains of *P. dorei*, *B. uniformis* and the strain of *P. distasonis* PtFD14MH1. The ability of different *Bacteroides* species and *P. distasonis* isolates, collected from a healthy donor for fecal microbiota transplantation (FMT), to adhere to the intestinal epithelial cells HT-29 and Caco-2 (non-mucus secreting cell) was recently reported [65]. The authors observed that the three isolates of *B. caccae*, and among the six isolates of *P. distasonis* only one was able to adhere to HT-29 and Caco-2 cells [65]. Our contrasting findings can be explained by the differences between the strains and the cell line tested (a mucus secreting cell).

In this study it was observed that the ability to invade HT-29-MTX cells was different between species and strains, where the highest ability to invade was achieved by *P. distasonis* PtFD14MH1 followed by *P. dorei* strains PtF D16P1 and PtF Sb8. The *B. uniformis* strains were not able to invade HT-29-MTX cells. A severe damage to the tight junctions of HT29-MTX cells after exposure to *P. dorei* strains and *P. distasonis* was observed, in contrast with the *B. uniformis* strains that cause a minor disturbance. To the best of our knowledge, this is the first study that reports the ability of *Bacteroides* species and *Parabacteroides* to invade mucus secreting cells. Different number of copies of the thiol-activated cytolysin (Listeriolysin O, LLO) of the subsystem of *Listeria* Pathogenicity Island LIPI-1 were identified in the genome of the *P. dorei* strains, *B. uniformis* and *P. distasonis* PtFD14MH1, except in the strain *P. dorei* PtFSb6. It is known that LLO, a pore-forming toxin is able to exert lytic activity in different types of cells, including the intestinal epithelial cell Caco-2 [73,74,75]. The presence of pore-forming toxins in the genome of *P. dorei* was recently described in the strain *P. dorei* CL03T12C01, including a Hly-III homologue [76]. The transepithelial electrical resistance (TEER) of Caco-2 cells decreases very rapidly (30s) after exposure to LLO at 1 μM [75]. A similar impairment of TEER values in the HT-29/B6 human colon cells was previously reported [77]. The decline in TEER values reported by Cajnko et al. [75] was associated to pore formation and to the disturbance of the tight junction protein claudin-1. Several bacteria, namely group A *Streptococcus* and *S. agalactiae* are able to increase intestinal permeability by disrupting tight junctions, which allows their paracellular passage [78]. The work of Vieira et al. [79] evidenced the ability of *Enterococcus gallinarum* to break the gut barrier being translocated to the liver, spleen and mesenteric lymph nodes, leading to the development of autoimmunity. The extent to which the thiol-activated cytolysins found in our strains contributes to the invasion of intestinal epithelial cells and disruption of tight junctions is one of our current research lines.

It was reported that the 1E6 human CD8+T cell clone, which intermediates the damage of β cells by recognizing the major HLA-A*0201-restricted, preproinsulin signal peptide (ALWGPDPAAA_15–24_, AWL) is able to recognize a massive number of different peptides [80]. Moreover, insulitic lesions in T1D patients show a significant occupancy with CD8+ T cells that recognize HLA-A*0201–ALWGPDPAAA [81]. The variants of the preproinsulin signal peptide AWL with sequence **MV**WGPDP**LYV** and **RQF**GPD**WIV**A were identified in *B. fragilis*/*thetaiotaomicron* and *C. asparagiforme*, and this latter variant was able to activate the 1E6 T cell with higher potency than the ALW sequence highlighting the potential development of autoimmunity by the T- cell cross-reactivity with bacterial derived peptides [49]. The results of the molecular mimicry search in the present study showed the presence of variants of the human natural preproinsulin ALW in the strains of *P. dorei*, *B. uniformis* and *P. distasonis*. It is important to highlight that *P. distasonis* PtFD14MH1 carries a variant similar to the one found in *C. asparagiforme* (**RRY**G**K**D**WIV**A); in contrast the strains of *P. dorei* and *B. uniformis* carry a variant similar to the one found in *B. fragilis*/*thetaiotaomicron* (**MV**WGPD**NFYV**). Intriguing, only the strain of *B. uniformis* PtFSb3P5 showed a variant of the human insulin B:9–23 peptide (HL**L**EALY**MTY**GE, SHL). The reported variant of the SHL in *P. distasonis* 33B (**RI**LVE**L**LYLVC**S**E**YL**) is able to promote an immune response to the endogenous insB:9–23, and the exposure of female NOD mice to *P. distasonis* mimic peptide during the gut microbiota maturing precipitated the onset of T1D [49]. Our findings, together with the others discussed, empower the hypothesis that common gut bacteria may injure the gut integrity, allowing for bacterial dissemination and eliciting an immune response that, in susceptible hosts, may trigger autoimmunity [82].

Several studies have reported the beneficial role of different *Lactobacillus* species on delaying the development of T1D or reducing its complications, using mice models [83,84,85,86]. In the current study the fecal samples of T1D children showed lower numbers of *Lactobacillus* spp. in comparison with control children, and such findings were also observed in fecal samples of Spanish T1D children [17]. Interestingly, we observed that the invasive ability of *P. dorei* and *P. distasonis* PtFD14MH1, using HT-29-MTX cells was impaired in the presence of *L. casei* DSM 20011, except for the strain *P. dorei* PtFD16P1. Probiotics (non-pathogenic microorganisms that are capable of protecting and promoting the health status of the host, such as *Lactobacillus* spp.) can act against pathogens through different mechanisms, namely by producing antibacterial substances like bacteriocins that prevent their replication, competition for scarce nutrients in the host, anti-adhesive and anti-invasive action, and competitive exclusion by obstructing the adherence of pathogens to host cell binding sites [87,88]. One of the great benefits that *L. casei* strains can possibly exert against the invasion ability of our tested strains of *P. dorei* and *P. distasonis* is its known capacity to promote the abundance of tight junction proteins and mucin [87,89]. The inability of *L. casei* DSM 20011 to inhibit the invasion of strain *P. dorei* PtFD16P1 requires further investigation.

The viability of HT-29-MTX-E12 cells was impaired by the exposure to the secretome of several *P. dorei* strains, the strains of *B. uniformis* and the *P. distasonis* PtF D14MH1. Recently the impact of the secretome of 157 gut bacteria on the growth rates of 5 colorectal cancer cell lines (CRC), including HT-29 was evaluated [90], and among these 157 gut bacteria, 12 belonged to the genus *Bacteroides* comprising the species *P. dorei*, *B. eggerthii*, *B. fragilis*, *B. massiliensis,* and *B. ovatus*. The effect of the *Bacteroides* species on cell growth of HT-29 cell line varied from an enhancement to a decline [90]. The negative impact of *Bacteroides* species on the growth of the CRC lines was attributed to the action of lipoproteins at the bacterial surface that have a high affinity for cobalamin, confiscating it from the culture medium, making this crucial vitamin inaccessible to the human cells, hence impairing their growth [90,91]. We can anticipate that besides the role of lipoproteins, other components of the secretome of our strains may be involved in the impairment of the viability of HT-29-MTX-E12 cells, namely the bacterial protease arsenal, which remains to be studied.

## 5. Conclusions

The recovery of *Bacteroides* species from the fecal samples of T1D and control children allowed us to expand our knowledge about their potential contribution to elicit adverse effects in susceptible hosts such as T1D children. The reason for the higher recovery of *P. distasonis* from T1D fecal samples demands additional investigation. It is noteworthy the observed invasion ability and capacity of some *P. dorei* strains and *P. distasonis* PtFD14MH1 to damage the tight junctions of the intestinal epithelial cells. The depletion of *Lactobacillus* spp. on the gut microbiota of T1D children was noticed, and its ability to inhibit the invasion ability of the tested strains was remarkable. These data together with other previous reports, evidence the continued efforts that have to be made to find the necessary balance between the gut microbiota of T1D children.

Our data also highlights the need to explore the contribution of the molecular mimicry, identified in the tested strains, to the development of autoimmunity, and offer new opportunities to find tools that can prevent and treat this disease that has such high prevalence and causes severe comorbities in medium to long term.

## Figures and Tables

**Table 1:** Genomic features of the analyzed strains.

Strains	Size (Mb)	Contigs	GC (%)	Genes	RNAs
*P. dorei* PtFD1P5	5880	153	42.2	5184	77
*P. dorei* PtFD8M1	5121	62	41.4	4295	76
*P. dorei* PtFD16P1	5588	101	42.1	4824	75
*P. dorei* PtFD16M14	5592	110	42.1	4835	77
*P. dorei* PtFSb6	5849	86	42.2	5164	78
*P. dorei* PtFSb8	5120	72	41.4	4288	74
*P. dorei* PtFC1P2	5479	83	41.9	4710	76
*B. uniformis* PtFD3Pch2	5457	8	44.1	4597	59
*B. uniformis* PtFSb3P5	438	21	46.4	3644	62
*P. distasonis* PtFD14MH1	5066	81	45	4240	85

**Table 2:** Sequences of the bacterial mimics for human insulin B:9–23 peptide (SHL) and human natural preproinsulin (ALW).

Human Insulin/Bacterial Peptide	Sequence	Identity	Reference
Human insulin B:9–23 peptide (SHL)	SHLVEALYLVCGERG	Wildtype	[48]
Variant of SHL	HL**L**EALY**MTY**GE	*B. uniformis* PtFSb3P5	This study
Human Natural preproinsulin (ALW)	ALWGPDPAAA	Wildtype	[49]
Variant of ALW	**MV**WGPDPLYV *	*Bacteroides fragilis*/*thetaiotaomicron*	[49]
Variant of ALW	**MV**WGPD**NFYV**	*P. dorei* and *B. uniformis* PtFD3Pch2	This study
Variant of ALW	**MV**W**S**PDP**LYV**	*B. uniformis* PtFD3Pch2	This study
Variant of ALW	**RQF**GPD**WIV**A	*Clostridium asparagiforme*	[49]
Variant of ALW	**RRY**G**K**D**WIV**A	*P. distasonis* PtFD14MH1	This study

* Bold letters represent amino acids that are different from the reference preproinsulin-derived sequence.

## Supplementary Materials

The following are available online at https://www.mdpi.com/article/10.3390/microorganisms9071436/s1, Figure S1: Growth in Brain Heart Infusion (BHI), Defined medium with glucose (DM GLU) and Defined medium supplemented with porcine gastric mucin (1%, *w*/*v*) (DM MUCIN), Figure S2: Cluster analysis by BOX rep-PCR (a) and ERIC rep-PCR (b) of *Bacteroides* species, *Phocaeicola dorei* and *Parabacteroides distasonis* isolated from T1D and Control children, Figure S3: Genome map of *P. dorei* PtFD1P5 (a), *P. dorei* PtF D8M1 (b), *P. dorei* PtF D16M14 (c) , *P. dorei* PtF Sb8 (d), *P. dorei* PtF Sb6 (e) *P.dorei* PtFC1P2 (f) and *B. uniformis* PtFSb3P5 (g), Figure S4: Phylogenetic relationships of the whole genome sequenced strains of *P. dorei*, *B. uniformis* and *P. distasonis* based on 30 genes, Table S1: Primers used in qPCR, Table S2: qPCR reaction conditions used for the tested bacterial groups, Table S3: Genes selected for MLSA, Table S4: Antibiotic susceptibility, Table S5: Minimum Inhibitory Concentration of Imipinem by ε test, Table S6: Identification of resistance genes in the genomes of *P. dorei*, *B. uniformis* and *P. distasonis* strains using the CARD/RGI, Table S7: SEED subsystem category distribution of the genomes of *P. dorei*, *B. uniformis*, and *P. distasonis* strains according to RAST annotation, Table S8: Identification of prophage sequences using PHASTER, Table S9: Identification of sequences of CRISPR/Cas in the analyzed genomes using CRISPRCasFinder.
