# Supplementary material for: Insights from Bacteroides Species in Children with Type 1 Diabetes
Source: Microorganisms. 2021 Jul 2;9(7):1436. doi: 10.3390/microorganisms9071436 (PMC8306409; doi:10.3390/microorganisms9071436)
Supplement: Supplementary file 1 [file microorganisms-09-01436-s001.zip › Figure S1.pdf]

Figure S1

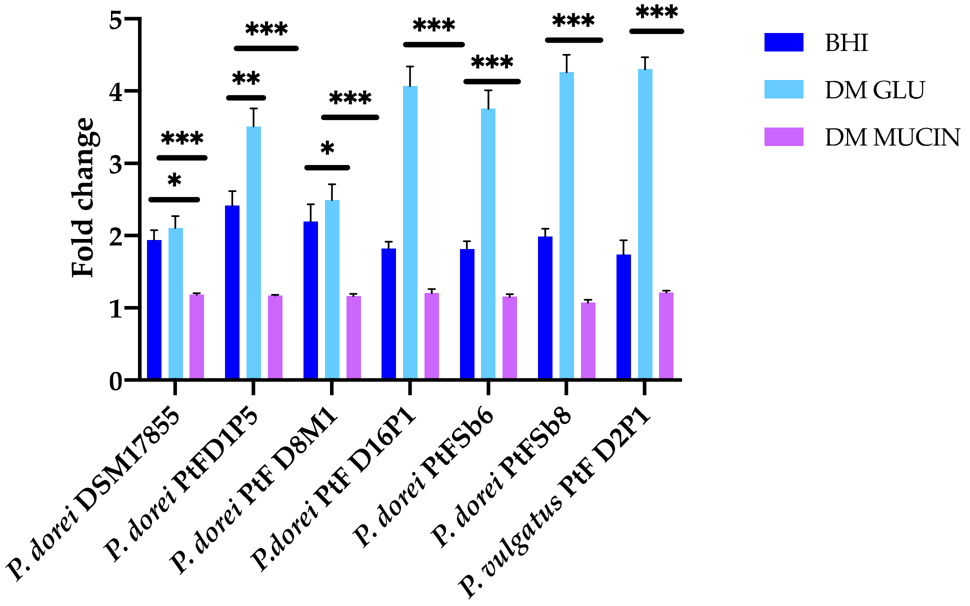

Figure S1. Growth in Brain Heart Infusion (BHI), Defined medium with glucose (DM GLU) and Defined medium supplemented with porcine gastric mucin (1 %, w/v) (DM MUCIN).
