# Supplementary material for: Insights from Bacteroides Species in Children with Type 1 Diabetes
Source: Microorganisms. 2021 Jul 2;9(7):1436. doi: 10.3390/microorganisms9071436 (PMC8306409; doi:10.3390/microorganisms9071436)
Supplement: Supplementary file 1 [file microorganisms-09-01436-s001.zip › Figure S2.pdf]

(a)

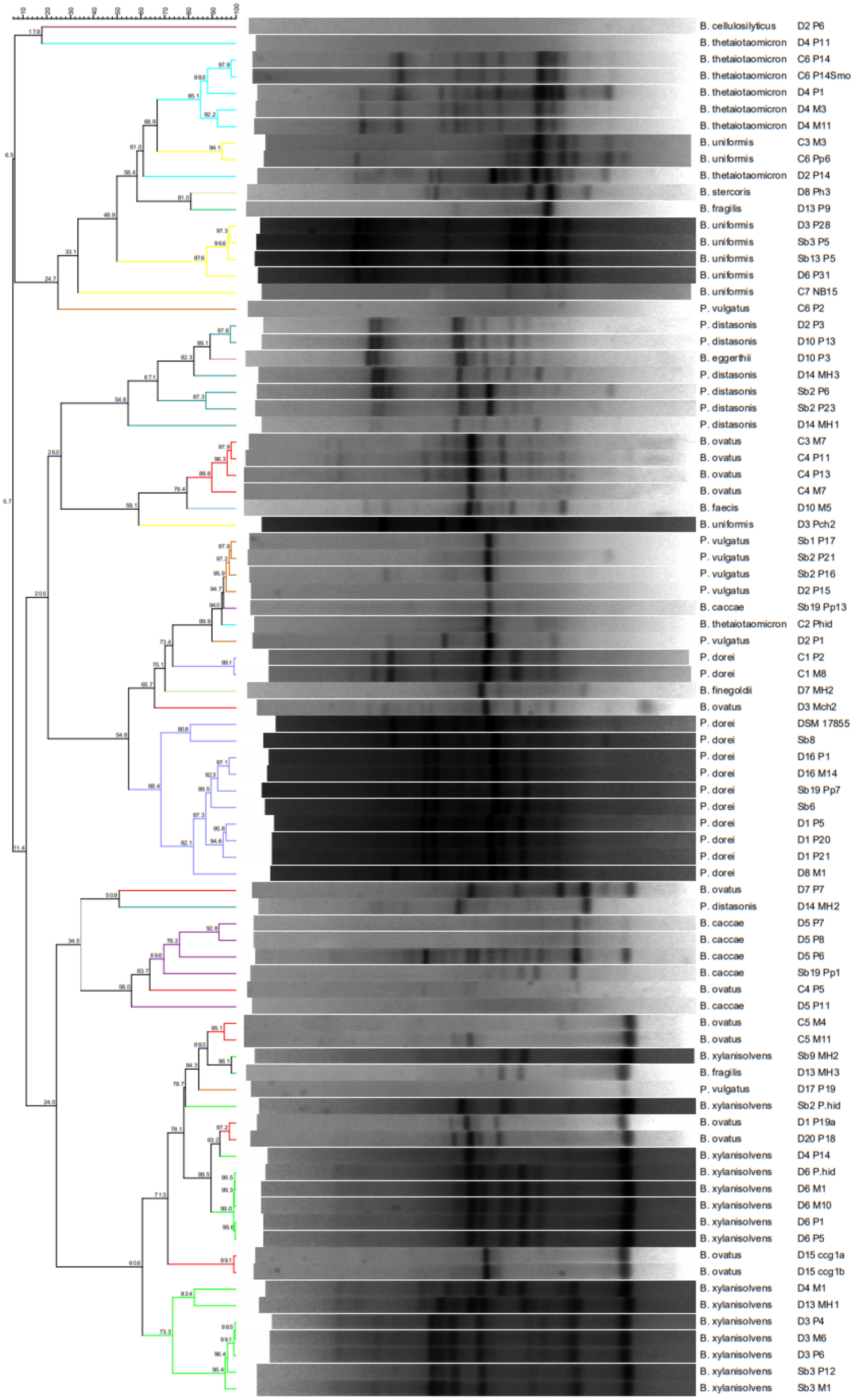

B. cellulosilyticus D2 P6
B. thetaiotaomicron D4 P11
B. thetaiotaomicron C6 P14
B. thetaiotaomicron C6 P14Smo
B. thetaiotaomicron D4 P1
B. thetaiotaomicron D4 M3
B. thetaiotaomicron D4 M11
B. uniformis C3 M3
B. uniformis C6 Pp6
B. thetaiotaomicron D2 P14
B. stercoris D8 Ph3
B. fragilis D13 P9
B. uniformis D3 P28
B. uniformis Sb3 P5
B. uniformis Sb13 P5
B. uniformis D6 P31
B. uniformis C7 NB15
P. vulgatus C6 P2
P. distasonis D2 P3
P. distasonis D10 P13
B. eggerthii D10 P3
P. distasonis D14 MH3
P. distasonis Sb2 P6
P. distasonis Sb2 P23
P. distasonis D14 MH1
B. ovatus C3 M7
B. ovatus C4 P11
B. ovatus C4 P13
B. ovatus C4 M7
B. faecis D10 M5
B. uniformis D3 Pch2
P. vulgatus Sb1 P17
P. vulgatus Sb2 P21
P. vulgatus Sb2 P16
P. vulgatus D2 P15
B. caccae Sb19 Pp13
B. thetaiotaomicron C2 Phid
P. vulgatus D2 P1
P. dorei C1 P2
P. dorei C1 M8
B. finegoldii D7 MH2
B. ovatus D3 Mch2
P. dorei DSM 17855
P. dorei Sb8
P. dorei D16 P1
P. dorei D16 M14
P. dorei Sb19 Pp7
P. dorei Sb6
P. dorei D1 P5
P. dorei D1 P20
P. dorei D1 P21
P. dorei D8 M1
B. ovatus D7 P7
P. distasonis D14 MH2
B. caccae D5 P7
B. caccae D5 P8
B. caccae D5 P6
B. caccae Sb19 Pp1
B. ovatus C4 P5
B. caccae D5 P11
B. ovatus C5 M4
B. ovatus C5 M11
B. xylanisolvens Sb9 MH2
B. fragilis D13 MH3
P. vulgatus D17 P19
B. xylanisolvens Sb2 P.hid
B. ovatus D1 P19a
B. ovatus D20 P18
B. xylanisolvens D4 P14
B. xylanisolvens D6 P.hid
B. xylanisolvens D6 M1
B. xylanisolvens D6 M10
B. xylanisolvens D6 P1
B. xylanisolvens D6 P5
B. ovatus D15 ccg1a
B. ovatus D15 ccg1b
B. xylanisolvens D4 M1
B. xylanisolvens D13 MH1
B. xylanisolvens D3 P4
B. xylanisolvens D3 M6
B. xylanisolvens D3 P6
B. xylanisolvens Sb3 P12
B. xylanisolvens Sb3 M1

(b)

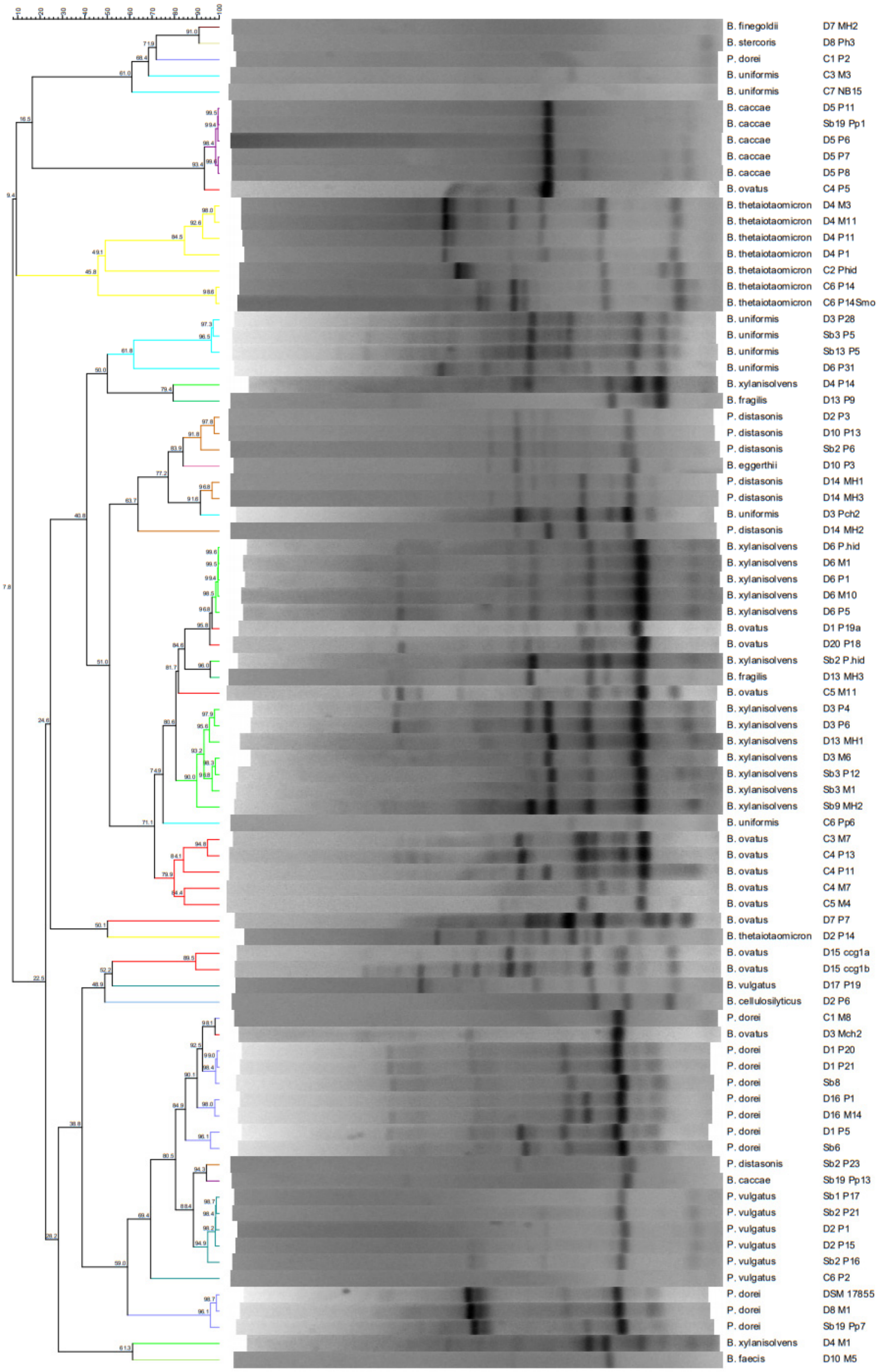

Figure S2. Cluster analysis by BOX rep-PCR (a) and ERIC rep-PCR (b) of *Bacteroides* species, *Phocaeicola dorei* and *Parabacteroides distasonis* isolated from T1D and Control children. Similarity values (%).
