# Supplementary material for: Insights from Bacteroides Species in Children with Type 1 Diabetes
Source: Microorganisms. 2021 Jul 2;9(7):1436. doi: 10.3390/microorganisms9071436 (PMC8306409; doi:10.3390/microorganisms9071436)
Supplement: Supplementary file 1 [file microorganisms-09-01436-s001.zip › Figure S3.pdf]

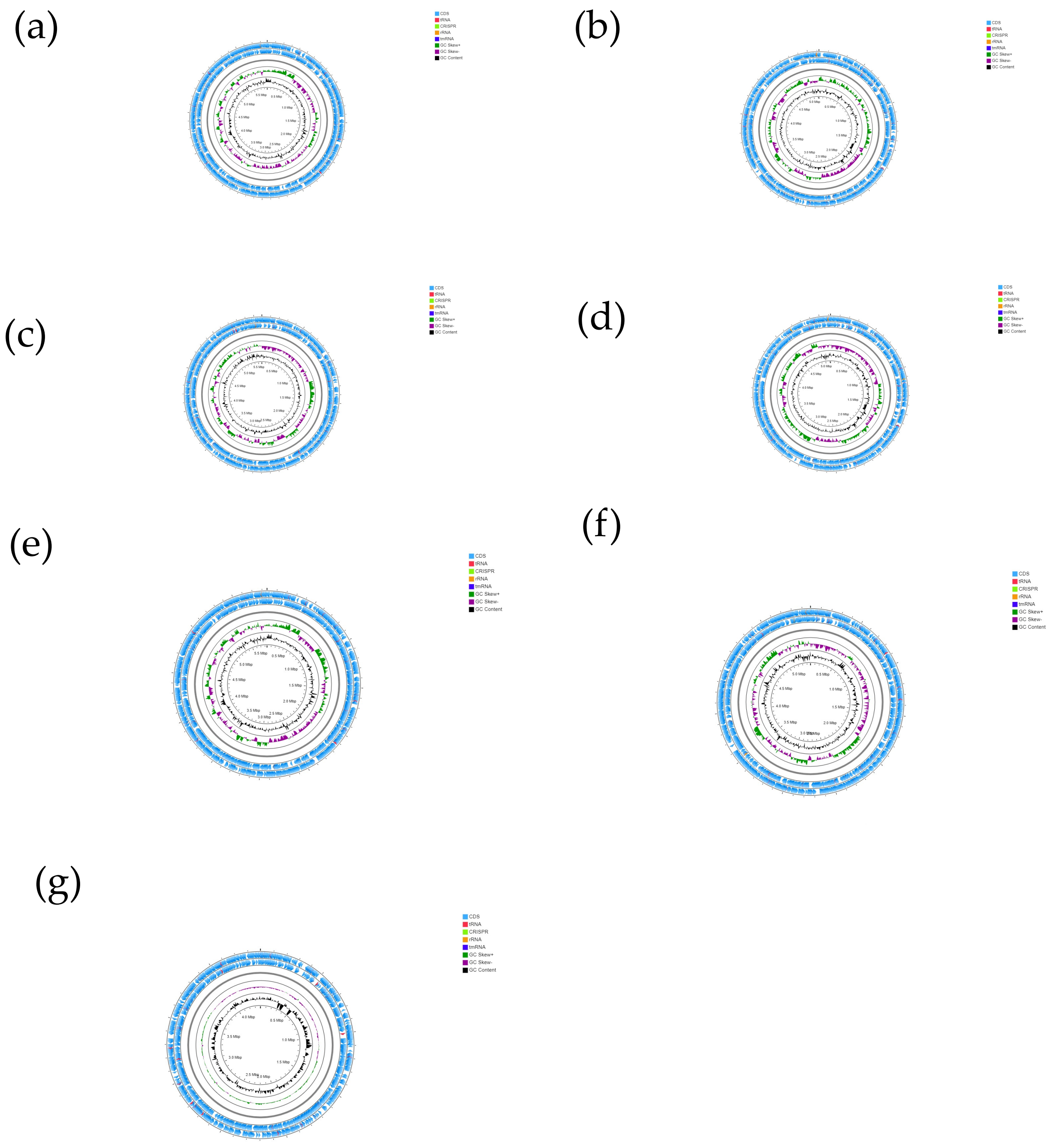

Figure S3. Genome map of *P. dorei* PtFD1P5 (a), *P. dorei* PtF D8M1 (b), *P. dorei* PtF D16M14 (c) , *P. dorei* PtF Sb8,(d) , *P. dorei* PtF Sb6 (e) *P.dorei* PtFC1P2 (f) and *B. uniformis* PtFSb3P5 (g). The outer most ring depicts the coding sequences in both clockwise and anti-clockwise direction. tRNA is depicted as red arrow heads, rRNA in orange and tmRNA in royal blue. CRISPR repeats regions are in lime. GC skewing is in green and purple and GC content in black.
