# Supplementary material for: Insights from Bacteroides Species in Children with Type 1 Diabetes
Source: Microorganisms. 2021 Jul 2;9(7):1436. doi: 10.3390/microorganisms9071436 (PMC8306409; doi:10.3390/microorganisms9071436)
Supplement: Supplementary file 1 [file microorganisms-09-01436-s001.zip › Figure S4.pdf]

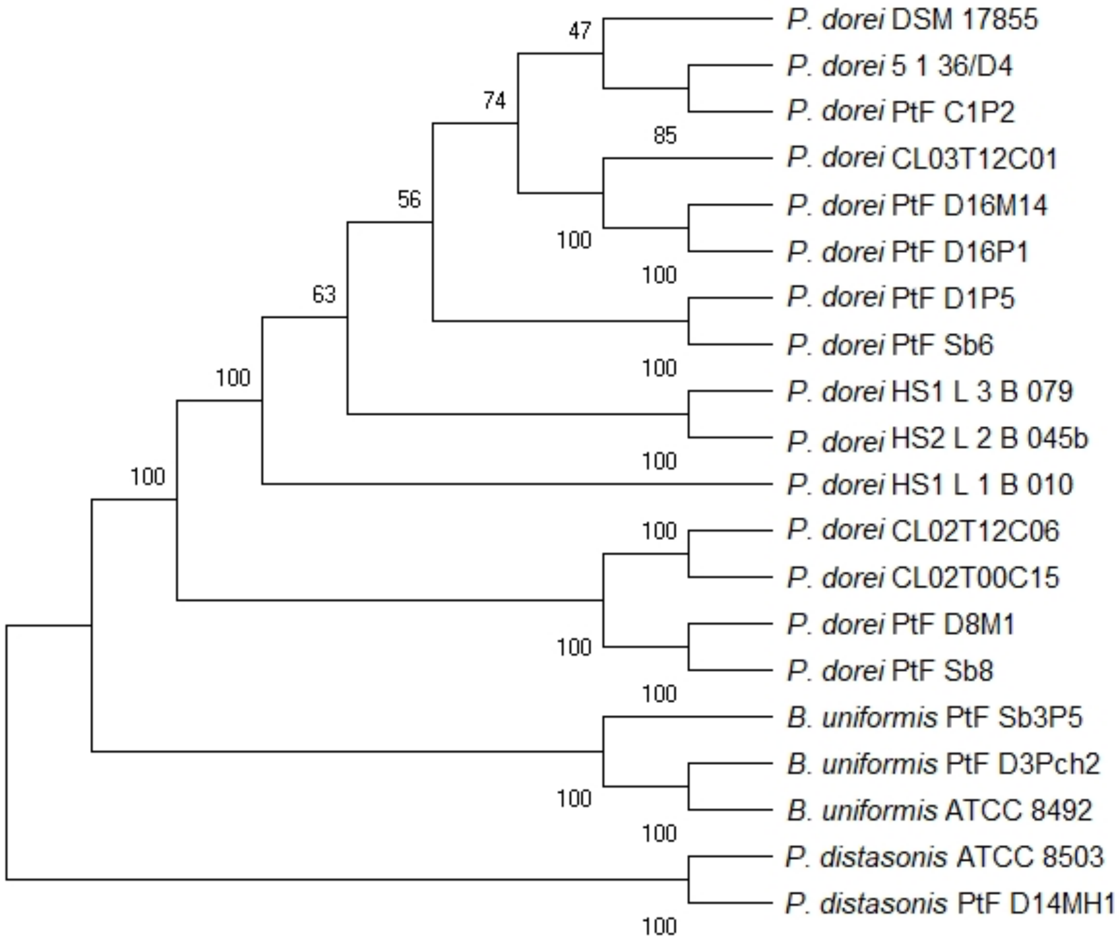

Figure S4. Phylogenetic relationships of the whole genome sequenced strains of *P. dorei*, *B. uniformis* and *P. distasonis* based on 30 genes. The dendogram was constructed using the UPGMA method. Numbers at nodes indicate the percentage bootstrap values of 1000 replicates. The sequences of the genes of the strains of *P. dorei* DSM 17855, CL03T12C01, CL02T12C06, CL02T00C15, HS1 L3 B 079, HS2 L2 B045b, HS1 L1B010, *B. uniformis* ATCC 8492 and *P. distasonis* ATCC 8503 were used as reference.
